# A perspective of massive open online courses (MOOCs) and public health

**DOI:** 10.3389/fpubh.2022.1058383

**Published:** 2022-12-14

**Authors:** Silvana Bettiol, Rhea Psereckis, Kate MacIntyre

**Affiliations:** ^1^Tasmanian School of Medicine, College of Health and Medicine, University of Tasmania, Hobart, TAS, Australia; ^2^Public Health Services, Department of Health, Hobart, TAS, Australia

**Keywords:** MOOCs, public health, education, professional, workforce, practice

## Abstract

Massive open online courses (MOOCs) have emerged as an innovative educational technology relevant to and affecting higher education, professional development, and lifelong learning. This paper introduces the principles of MOOCs and reviews the development of these platforms over time. We reflect upon the considerable investment by institutions to develop, deliver and promote such courses, particularly in public health. While open to interpretation, the inherent power, influence, and effectiveness of MOOCs is unquestionable. The potential contribution of MOOCs to public health education is immense, with almost universal reach and access. However, apart from research into participant engagement and knowledge, MOOC-related research and evaluation continue to lag with the rapid proliferation of these courses in response to emerging challenges, as seen with the Coronavirus Disease 19 (COVID-19) pandemic. This makes analyzing the contribution of MOOCs to public health education, health promotion and community programs challenging. This perspective article provides a robust rationale for the necessity of MOOCs and their utility in upskilling health professionals and the general public. It builds on current knowledge to comprehensively explore the factors influencing the development, and application of MOOCs.

## Introduction

Public health is an interdisciplinary field concerned with understanding and influencing the population's health and wellbeing. While public health professionals assume various roles and responsibilities, the workforce must profoundly understand population health, including the social and ecological determinants of health. Public health education seeks to advance knowledge of public health evidence and interventions and aims to develop skills to address public health issues and influence change at a population level. Most public health schools offer interdisciplinary and multidisciplinary programs to support career preparation therefore drawing on and developing partnerships with non-traditional public health disciplines is necessary to tackle complex problems, with recent examples from engineering in humanitarian action (1) and urban health (2). However, there is considerable variation in public health competency frameworks and curricula content worldwide (3). This is reflective of geographic and cultural variability and the responsiveness of educational institutions and governments to factors impacting public health globally. By leveraging the efficiencies in content delivery, massive open online courses (MOOCs) can promote awareness of public health challenges as well as global issues. According to Meinert et al. (4), MOOCs may be a sustainable method for increasing citizen engagement in global concerns and health-related issues devised around health behavior research. Organizational MOOCs aimed at increasing knowledge and self-management of health issues can potentially improve health promotion and literacy (5, 6). High-quality content can be delivered to a large audience and updated with evidence from the latest scientific studies, which is not always possible with face-to-face instruction.

The Coronavirus Disease 19 (COVID-19) pandemic has stretched many health services across the globe. Given the rising costs of health and social care systems in many countries (7, 8), there is a need for new, improved, and accessible web-based resources and information to assist with health prevention and care. The flexible MOOC platform complements the increasing move to working from home, supported by many organizations and sectors since the COVID-19 pandemic. In addition, MOOCs provide on-demand learning opportunities in a rapidly changing workplace (9), making them attractive to small organizations that may lack the scale to invest in formal in-house training (10). The personal and professional gains are self-evident from MOOC participation and are well-described by authors such Blum et al. (11) and Chesniak et al. (12). We recognize the need for more rigorous research on MOOCs and their effectiveness and intended outcomes in the public health context. The aim of this perspective article is to review the current application of MOOCs across various disciplines and consider emerging trends and future potential for MOOCs in public health education.

### The evolution of MOOCs

The original aim of the MOOC was to offer a self-organized learning experience in a field of study facilitated by experts in the area (13). It was introduced over 20 years ago as a revolution in education for its “disruptive content delivery method” by testing new pedagogical approaches and technological tools (13). MOOCs addressed potential barriers to educational engagement, by offering free access, and flexibility through their information and communications technologies (ICT)-based structure (14, 15). A course could include a few participants to as many as several thousand, actively engaging at their convenience (16). Learning on an online platform represented an alternative to face-to-face instruction while still offering collaborative learning with interactive forums. MOOCs have since grown steadily in popularity, driven by expanding internet access (17, 18). In 2019, an estimated 110 million individuals enrolled in an online course (19, 20), doubling to 220 million in 2021 (21). Consequently, millions of learners have engaged in non-traditional learning platforms promoting their new skills by signaling these learning successes to a broader community.

Since their inception, MOOCs have evolved into two types of courses: the Connectivist MOOC (cMOOC) and the Extended MOOC (xMOOC) (22). The main focus of cMOOCs is the creation and generation of knowledge, whereas the xMOOCs are content-based and focus on disseminating knowledge (23). Variations exist around the delivery of knowledge and skills development, depending on the discipline. While conceptual and quantitative descriptive studies of MOOCs in the disciplines of education, computer science, and engineering dominate, the educational approaches are practice-based and readily converge with the science and art of public health (24). For example, branches of public health, such as epidemiology, lend themselves to independent study. In contrast, other public health branches, such as biostatistics, which are simulation-based or have short answers, may use machine-scoreable assessments (25). Some MOOCs are designed to create an authentic learning experience by allowing participants to develop projects or blogs with other students to form social learning groups (26). McAuley et al. (14) proposed that the MOOC model would provide an ecosystem for exploring how individuals may develop the knowledge, skills, and attitudes they need to thrive in an economy. The model would also allow individuals the opportunity to join the community of practice at their own pace (16). This vision reflected the pedagogical principles of professional development, independence, and lifelong learning.

### MOOCs in public health education

Many universities and colleges worldwide use MOOCs to provide a “flipped classroom” model for improved learning (27, 28). This approach has seen MOOCs gain traction in healthcare education, including medicine, pharmacy, and nursing (29–32). The MOOC technology has been a welcome method to supplement and augment medical training and inter-professional learning, which was necessary during the COVID-19 pandemic (33, 34). The COVID-19 pandemic has increased the demand for and provision of public health education.

The numerous Australian, US and UK universities that supported the emergence of MOOCs continue to provide central coordination and multiple MOOC offerings (35). The Organization for Economic Co-operation and Development (OECD) noted that the introduction of MOOCs undoubtedly generated a potential alternative pathway to traditional higher education qualifications (36). They have also shown to be an impetus for innovation, facilitating new approaches in education, including healthcare, by allowing diverse organizations to enter the higher education market (37). Figures from the MOOC aggregator site “Class Central” indicate that more than 900 universities globally launched free online courses using popular platforms such as FutureLearn and Coursera in 2021, with many governments launching their country-specific MOOC platforms (38). However, substantial variations of content and complexity exist between these platforms, particularly in course specialization and degrees and certificates awarded for courses (39).

A review of MOOCs in health education during the COVID-19 lockdown in 2021 by Dolores et al. (40) reported that 117 MOOCs, delivered by different platforms, were targeted at the general population, with the majority aimed at health promotion, food and nutrition, and mental health. Content delivery using MOOCs could provide a flexible modality to reflect the dynamic nature of public health if locally relevant (41). In addition, the potentially high number of course participants presents an opportunity to bring together a uniquely diverse group of people, allowing course cohorts to obtain a global and systems perspective which is an important component of public health education (42, 43). We note limited research examining the value of MOOCs as a platform for the delivery of public health education, reflected by a paucity of literature other than the reporting the numbers of participants.

### Future trends of MOOCs in public health education

The earliest MOOC developments in public health education were recorded by the Johns Hopkins Bloomberg School of Public Health (JHSPH) during its 2012–2013 academic year. The school's decision to offer MOOCs was based on its already extensive experience with online and open education (44). This innovative approach served as a marketing element for JHSPH, offering a taster experience for a full degree program and attracting those ready and able to pursue formal coursework. In addition, it developed the potential to forge new partnerships with other universities and organizations, highlighting the growing interest in public health outside traditional degree programs.

MOOCs have materialized as effective tools for scaling-up education (45). McAuley et al. (14), viewed MOOCs as a reflection of a society where citizens are active agents in knowledge creation and dissemination processes. This implies that “digital citizenry” reflects the ability of citizens to connect, innovate, and reconfigure the known into new knowledge (14). MOOCs can also complement more traditional courses by simultaneously propagating information to many participants. McAuley and his team (14) had foreseen that training and educating large numbers of people in digital learning would be effective tools to produce change on a large scale. MOOCs offer a platform to support the tenet of public health promotion, which endeavors to connect people to improve health and wellbeing.

The personalization of MOOCs is a growing trend aimed at improving learners' individual experiences (46). Researchers such as Lambert (47) note that many organizations and educators have created public health programs for disadvantaged groups and communities to provide supportive, equitable, and targeted education. Examples include research training for health professionals in low- and middle-income countries (LMICs) (48) and One Health education in Kakuma refugee camp in Kenya (49). MOOCs can be directed to help the most vulnerable, such as the “Talk-to-Me” MOOC intervention for suicide prevention and mental health education among tertiary students (50). MOOC content developed in collaboration with stakeholders, a design process coined as co-creation, offers a viable method of developing course material tailored to the needs of a specific population group, such as the MOOC to Improve Digital Health Literacy in Pregnant and Lactating Women (51). Digital health literacy (DHL) has been referred to as a “super social determinant of health,” (52) and the use of MOOCs to improve DHL skills of health care professionals (53) can be an effective strategy, with an opportunity to employ the process of co-creation in its design and development (54).

Targeted training outside traditional degree programs facilitates higher efficiency for higher education systems, including continuing professional development programs (19). This often fulfills labor markets' needs and improves economic diversification and competitiveness by retaining workforce (9, 55). For example, during the COVID-19 crisis, universities offered new micro-credentialing in public health for school-based professionals (56), and the Imperial College of London School of Public Health delivered MOOCs that were freely available to all NHS staff (57). MOOCs created to support skill development have proven to be popular, with higher enrolment and completion rates for courses that target work-related skills (58, 59) compared to the low completion rates of courses with more general content (60). However, the limited research in this area suggests the true effect of such credentials in various labor markets is unknown (61). Nevertheless, digital badges, micro-credentials, and certificates are promoted as an alternative to the more traditional credentials offered by higher education institutions (19). Fees associated with the more advanced MOOCs that offer certificates and micro-credentialing appear to be a significant departure from the original aim of MOOCs. Yet these changes have extended the reach of traditional higher education institutes to new students across the globe (62). Despite the challenges and complex nature of content, design and delivery of public health MOOCs, the desire to share knowledge and expertise is evident. We summarize our pearls and pitfalls in Table 1.

**Table 1:** The pearls and pitfalls of MOOCs in public health.

	**Pearls**	**Pitfalls**
Course delivery	• Enables greater learner participation that is not constrained by physical space. • Platform available to use by a range of providers such as educational institutions, private organizations, and governments. • May be free or low cost to the learner to commence. • Online delivery method more resilient to external challenges e.g., pandemics	• Requires reliable internet access. • Potential for significant costs to the MOOC provider to develop and maintain the course. • Often some cost to the learner to get certification on course completion. • High attrition rates. • May be challenging to moderate where large numbers of learners enrolled. • Difficult to adequately grade. • Lack of research to address pedagogical limitations.
Course participation	• Can be made accessible to a wide audience or tailored to an audience or organization. • Increasing number of MOOCs provides choice for learners to meet interests or needs. • Brings people together in an online learning space.	• Wider participation may make tailoring course content to the audience more challenging. • With an increasing number of MOOCs available, providers may need to consider marketing strategies. • Volume of available MOOCs may create challenges for learners to find the “best” MOOC to suit their interests or needs.
Course content	• Online format allows content to be updated easily. • Agility to adapt course content in response to emerging challenges. • Wide breadth of potential content. • Supports integration of content from across disciplines. • Flexible format that allows learners can progress and complete at their own pace.	• Requires regular maintenance to ensure content remains current. • Potential challenges in sourcing appropriate academic and technical expertise to inform course content and reach minimum quality standards. • Lack of agreed methodologies to evaluate potential impact, especially in the context of emerging public health challenges.

### MOOCs for public health professionals

Health professionals increasingly access MOOCs to support their professional development (63). MOOCs offer a platform for high-quality reskilling and upskilling opportunities, particularly those targeted at continuing professional education (64) as well as supporting job seekers to attain sustainable employment opportunities. Globally, public health systems play a crucial role in addressing workforce retention by preparing for evolving capability needs (65), and continuing education opportunities for the public health workforce are essential. For example, MOOCs have leveraged access to education by healthcare workers in many LMICs, where they lack access to quality improvement training and basic skills in healthcare delivery (66, 67).

The interdisciplinary field of public health does not have a single preferred training modality (68), therefore, the scope and modality in MOOC preferences are influenced by design, delivery, and organizational support. Archer et al. (68) found that programs could influence the engagement of full-time employees by designing courses with clearly articulated purposes and benefits. The public health workforce was generally more engaged in completion if the course certification was aligned to competencies; the course content had practical application and was shorter, allowing them to complete the course quickly. Universities around the world reported high response rates to interest in public health and the necessity to gain professional public health skills. The impact of the COVID-19 pandemic on employers left many to reconsider their training choice and mode of upskilling and reskilling (69–71). For many countries, providing public health information and upskilling healthcare workers to all disciplines was necessary, as the COVID-19 emergency response required a surge in staff for pandemic preparedness, education, planning, logistics, and operations (72–74). The pandemic has been a powerful test of the online learning model and of organizational ability to respond quickly and effectively by expanding the range and delivery of training programs, from undergraduate programs to community-based health promotion education (38, 75).

### Local public health MOOCs–An example from Tasmania

The COVID-19 pandemic challenged health systems globally, requiring them to respond with an integrated, multidisciplinary approach to preparedness, prevention, and management. Despite a whole of government response, Australia's federal division of power allowed policy formulation and innovation to occur at the state level, reflecting the impact of the pandemic for local communities. Tasmania's COVID-19 response aimed to contain the virus, minimize transmission, and protect vulnerable people and groups. This necessitated a range of evolving measures, including lockdowns and restricted travel, which had significant consequences on individuals and communities, including economic, social and psychological impacts.

The island state of Tasmania is isolated from the rest of Australia, with a limited public health workforce. It soon became evident that building a robust, skilled workforce was essential to an effective response. To advance existing expertise, close skill gaps and build surge capacity, creative solutions were explored to target and engage a large audience and deliver online training. As a result of agile leadership, a public health MOOC was developed in collaboration with the University of Tasmania and Public Health Services, Department of Health Tasmania (76). This MOOC aimed to provide initial training in contact tracing and active surveillance to support the development of a surge-ready workforce in Tasmania. It included an introduction to COVID-19, a summary of its epidemiology and prevention, and an overview of the public health response in Tasmania, including local systems and processes. These skills were applied through case studies and mock contact tracing interviews.

### Global public health MOOCs

One of the most successful MOOCs during the COVID-19 pandemic was delivered by the World Health Organization (WHO) through their interactive platform, OpenWHO (77). OpenWHO was developed by the WHO Health Emergencies Programme in partnership with Hasso Plattner Institute (HPI) in 2017 (78). It offered a range of life-saving content on various disease outbreaks and general material for frontline responders, healthcare workers and the general public. Before the COVID-19 pandemic, OpenWHO provided 18 different disease courses; by 2021, there were 38 courses focused on the COVID-19 response alone (79). Additionally, the number of learners increased from 140,000 users in 2019 to 7 million enrolments, with a significant increase in female engagement and diversity in the geographical location and user characteristics (80).

The OpenWHO course creation process is unique, devised to be current and up-to-date with acute health emergencies, with WHO technical experts providing resources and guidance online in real-time (81). Course material is free, with unlimited access, and delivered in low-bandwidth formats accessible *via* mobile or desktop devices. In addition, OpenWHO courses are offered in multilingual formats and local languages of communities affected by particular outbreaks, including one in sign language (82). George et al. (83) acknowledge that this has facilitated “the rapid, global sharing of life-saving knowledge to the audiences who need it, when they need it, in formats and languages they can understand.”

Similarly, other United Nations (UN) agencies and programs responded to the COVID-19 pandemic with innovative learning solutions for individuals, organizations and institutions *via* its many platforms. UN agencies and programs produce various courses in partnership with numerous organizations, with courses offered through different platforms, as seen through the work of The United Nations Institute for Training and Research (UNITAR) and United Nations Information Centers(UNRIC) (84, 85). The benefit of partnership-based programs is the possibility of combining discipline experts in training and development with experts in substantive areas and local contexts. Courses are accessible to the public and professionals in different languages and free of charge. Some courses run several times a year, and a few provide certification. Concurrently, the partnerships provide a mechanism to deliver a broad range of contemporary public health content, including sustainable development and human rights, climate change, nutrition and food security, young people, and their mental health (84, 85).

## Conclusion

Open online learning courses have grown substantially in recent years, with millions of new users enrolled (18). Class Central recorded 194,000 listed courses in the past 10 years, with micro-credentialing and MOOC-based degrees doubling in numbers from previous years (18). Our local experience of the COVID-19 pandemic demonstrated the opportunity to be agile in our response and adapt courses to local contexts and emerging needs, aligning them with participants' needs and experiences, and technological and financial resources. MOOCs developed in response to emerging challenges in public health require content to be regularly evaluated, updated, and improved. We have outlined the complex nature of content, design and delivery of public health MOOCs; however, it is evident that there is a need for agreed methodologies and guidelines that support robust evaluation to ensure homogeneity between studies. This includes standardization of reporting and process of data and a focus on evaluating outcomes, including knowledge, competencies and skills to examine MOOCs' benefits, effectiveness and impact in public health. More targeted research is required to better understand the role of MOOCs in public health education and health improvement to maximize their potential impact, especially in the context of emerging challenges.

## Data availability statement

The original contributions presented in the study are included in the article/supplementary material, further inquiries can be directed to the corresponding author.

## Author contributions

SB and KM contributed to the conception and design of the article. SB wrote the first draft of the manuscript. KM and RP wrote sections of the manuscript. All authors contributed to manuscript revision, read, and approved the submitted version.
